# Evaluation of Measurement Tools for Tobacco Product Displays: Is there an App for that?

**DOI:** 10.3934/publichealth.2015.4.810

**Published:** 2015-12-11

**Authors:** Todd B. Combs, Sarah Moreland-Russell, Jason Roche

**Affiliations:** Center for Public Health Systems Science, George Warren Brown School of Social Work, Washington University in St. Louis, St. Louis, MO

**Keywords:** tobacco product display, point-of-sale policy, tobacco control, retail setting, measurement

## Abstract

Tobacco product displays are a pervasive presence in convenience stores, supermarkets, pharmacies, and other retailers nationwide. The influence that tobacco product displays have on purchases and tobacco product initiation, particularly on young people and other vulnerable populations, is well known. An objective measurement tool that is valid, reliable, and feasible to use is needed to assess product displays in the retail setting. This study reports on the relative accuracy of various tools that measure area and/or distance in photos and thus could be applied to product displays. We compare results of repeated trials using five tools. Three tools are smartphone apps that measure objects in photos taken on the device; these are narrowed down from a list of 284 candidate apps. Another tool uses photos taken with any device and calculates relative area via a built-in function in the Microsoft Office Suite. The fifth uses photos taken with the Narrative Clip, a “life-logging” wearable camera. To evaluate validity and reliability, we assess each instrument's measurements and calculate intra-class correlation coefficients. Mean differences between observed measurements (via tape measure) and those from the five tools range from just over one square foot to just over two square feet. Most instruments produce reliable estimates though some are sensitive to the size of the display. Results of this study indicate need for future research to test innovative measurement tools. This paper also solicits further discussion on how best to transform anecdotal knowledge of product displays as targeted and disproportionate marketing tactics into a scientific evidence base for public policy change.

## 1. Introduction

Tobacco product displays are a ubiquitous part of the retail landscape, found in convenience stores, supermarkets, pharmacies and other retailers. The tobacco industry spends over $750 million annually on product placement in the retail environment[1]. Product displays are often located in aisles and on counters, fully accessible to customers, or behind the counter where products remain visible. Tobacco companies compete for shelf space and representatives visit retailers frequently, offering taller and larger displays as they compete for greater product visibility[2]. When large numbers of tobacco products are placed side-by-side, they create a power wall that becomes a form of advertising[3],[4]. The pervasiveness of tobacco product displays has disproportionate implications for vulnerable populations, including adolescents, low income, and minority communities are exposed to a wide variety of tobacco products and advertisements[5]–[12]. For instance, youth in neighborhoods with higher tobacco retailer density are more likely to initiate smoking than those in neighborhoods with lower tobacco availability, and higher tobacco retailer density observed in neighborhoods with higher percentages of minorities is related to higher smoking rates in these populations[13]–[15].

Building an evidence base of tobacco industry product placement practices is critical to support policy interventions at the point of sale (POS). The development of methods to measure and compare sizes of tobacco displays is necessary to better understand the impact they have on vulnerable populations. The identification of reliable and valid tools to measure them would allow for widespread data collection and help to raise awareness among the public and ultimately, this evidence could serve as a basis for policies that restrict tobacco product displays to diminish exposure and reduce tobacco-related disparities[5].

Few studies have attempted to measure tobacco product displays systematically and instead have focused on the sizes of advertisements. Most of these have used subjective measurement methods like a visual scale ranging from “no advertising”, to “advertising covering most of the store”, or “in your face” advertising[16],[17]. One study included the amount of shelf space dedicated to cigarettes as a measure of advertising. Shelf space was measured by observers who counted the number of cigarette pack-facings to measure the magnitude of tobacco product displays but width and height were not calculated and measurements did not include non-cigarette tobacco products[8]. A similar study analyzed digital photos using Windows Photo Gallery software to identify the total number of visible tobacco packs, pack size, and product type[18]. A systematic review in 2013 found that of 88 store audit studies, the majority of which were designed to describe the content and prevalence of POS tobacco marketing or identify disparities, none of the measures and methods employed sought to capture the size of product displays, rather they counted the number and types of displays. Very few studies in the systematic review reported the reliability and validity of audit measures[19].

Mobile phones, wearable cameras, and other new technology, such as smartphone apps, are beginning to be evaluated for other purposes in health research. A comparison of 23 smartphone apps designed to perform opioid conversion for medical professionals identified wide inter-app variability in conversions and noted the need for improvements in accuracy and reliability[20]. An evaluation of the accuracy of 14 smartphone sound measurement apps found some apps to be accurate and reliable enough to assess occupational noise exposure[21]. A study of 98 smoking-cessation apps for Android and iPhone noted the increasing availability and use of apps for cessation and indicated that across both platforms, the apps lacked the recommended evidence-based practice, but if improved, could represent a promising cessation strategy at the population level[22]. Especially given the increasingly diverse functionality and adaptability of smartphone apps, new technology may hold promise for measuring the size of tobacco product displays. This paper provides results from tests of smartphone apps and other technologies that hold potential to help create an evidence base of tobacco product placement in the retail setting.

Specifically, we identify and test the reliability, validity, and feasibility of tools for measuring the size of retail tobacco product displays.

## 2. Methods

For this experimental study, we investigated innovative ways of measuring tobacco product display areas using photographic tools and methods. The study was deemed non-human subjects research and thereby exempted by Washington University's Institutional Review Board. Our selection criteria for inclusion of measurement instruments included 1) widely accessible, 2) easy-to-use, 3) conducive to in-store use, and 4) valid and reliable systems of measure. We considered various smartphone apps, the Narrative Clip wearable “life-logging” camera, and a feature of the Microsoft Office suite that measures shapes. Though with its price of $279, the Narrative Clip did not satisfy the first criterion of wide accessibility, the discretion allowed by the small device that clips on a shirt made it a candidate for unobtrusive in-store use[23]. Many people are familiar with the Microsoft Office suite of products, and most public and private organizations provide access to the software. A majority of Americans now own smart phones[24], and researchers, the general public, and members of the smallest grassroots tobacco control partners are increasingly familiar with smartphone technology.

Our primary criterion for selecting apps was for them to be compatible with at least one of the two most popular smartphone operating systems, iOS (Apple) and Android (Google)[25]. We began in Fall 2014 by broadly searching the iTunes App Store and Google Play for iPhone and Android apps that claimed to measure objects. In the iTunes App Store as well as the Google Play Store, preliminary searches for the term “measure” returned thousands of results. Eventually we narrowed the results by adding the terms “height” and “width” to the search. In iTunes, searching for the group of words (“measure”, “height”, and “width”) returned 32 apps and in Google Play it returned 252 apps. We then read the descriptions, reviews, and other available materials for each of the 284 apps and ruled out those that did not perform the necessary measurements. Only about 11%—13 out of 32 and 18 out of 252 in iTunes and Google Play respectively—were identified as potential candidates meeting the basic criteria of being able to estimate both height and width of objects.

The remaining 31 apps were downloaded. We immediately excluded those apps that only performed measurements “on the fly” without a way to save the calculations. Using these types of apps would require taking pen and paper to manually record each measurement while inside the store. Other apps were excluded based on poor operation upon download. Of the downloaded and working apps, only three allowed for dimensions to be calculated from a photo after leaving the store: EasyMeasure (iOS, $5.99), Partometer 3D (Android, $1.99), and ON 3D CamMeasure (Android, free). The EasyMeasure app allowed for calibration based on user height and the two Android apps measured object size via a reference object (of known dimensions) in the photo. In all, we tested three apps, the narrative cam, and Microsoft Office as instruments for measuring product displays at the point of sale.

**Figure 1.**
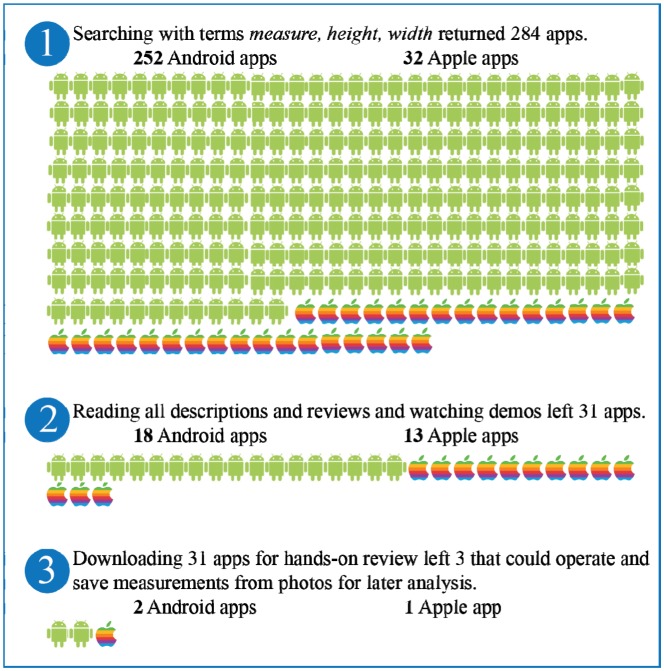
App selection process

We used a standard cigarette pack measuring 7.6 in^2^ (0.05 ft^2^) as a reference object for Microsoft Office, Partometer 3D, ON 3D CamMeasure, and Narrative Clip photos. The process for the reference object apps entails fitting a box on the screen around the object of known size (i.e., the cigarette pack) entering those measurements, and then fitting another box around the entire display. The app then uses the two onscreen measurements and the one known actual measurement to calculate the dimensions of the display. We imitated this process with the “insert shapes” function in Microsoft Office for photos taken with the phone cameras and the Narrative Clip camera. The EasyMeasure app uses the height of the device input by the user, an onscreen viewfinder to identify the point where the wall and floor meet, and geometric formulas to calculate distances, widths, and heights.

Before conducting the measurement trials, two testers, each using an iPod Touch (fourth generation) and a Samsung Galaxy S4 for the apps, the Narrative Clip, and PC versions of Microsoft Office, spent at least five hours becoming familiar with each method, taking photos, practicing calculations, and discussing challenges encountered. For the trials, 10 mock product displays measuring from two to 62 square feet were set up at the Center for Public Health Systems Science at Washington University. The testers took photos of each display and calculated measurements with each of the five instruments for a projected total of 100 trials.

## 3. Statistical Methods

For each instrument, measurement, and tester, we calculated validity and reliability estimates. To calculate validity of the measurements, we compared the results from each method to the actual physical dimensions, as measured by a tape measure. Each display was measured at least twice with a tape measure to ensure accuracy. To determine whether the actual size of the various displays influenced the measurements, we executed ordinary least squares regression models for each of the instruments and its measurements. Separately, and based on the distribution of differences between each instrument's measurements and physical measurements, we used Chebyshev's Inequality to predict the accuracy of each instrument. To estimate reliability, we calculated coefficients of inter-rater reliability between the two testers as well as intra-class correlation for each instrument's measurements and the physical measurements obtained with a tape measure.

## 4. Results

**Figure 2.**
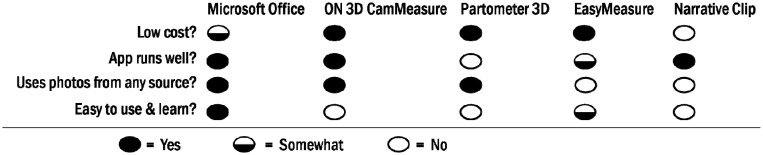
Relative usability of instruments

Throughout the training and test trials of the instruments, we developed pros and cons for each (summarized in Figure 2). The results below are organized by the main criteria for selection including accessibility and ease of use, validity, and reliability of the measures.

### 4.1. Accessibility and ease of use

Besides the Narrative Clip wearable camera, all of the instruments were relatively low cost and widely accessible for tobacco control partners and researchers. Two apps, Partometer 3D and EasyMeasure were prone to crashes. No technical challenges were encountered for the other three instruments.

An advantage for Microsoft Office, ON 3D CamMeasure, and Partometer 3D was that the photos could be imported from any device, but EasyMeasure only worked with photos taken in the app. Previous familiarity with Microsoft Office helped to make drawing boxes around objects in photos relatively easy. The smaller size of iPod and Android smartphone screens for use with the three apps presented challenges for manipulating the guides and measurement tools; the Partometer 3D and ON 3D CamMeasure were extremely sensitive to the touch, making measurements in these two apps the most time-consuming of all the instruments. The Narrative Clip takes photos every 30 seconds automatically, alternatively one can tap the front of the device photos at any time. Even with this capability, of the 20 attempts with the Narrative Clip, only 12 produced usable photos, as aiming the device and ensuring inclusion of entire displays was challenging without a lens through which to view the target area of the photo. Because of the required aiming and tapping, we concluded that the Narrative Clip could not be easily used as a “covert” option for in-store use.

### 4.2. Validity

**Figure 3.**
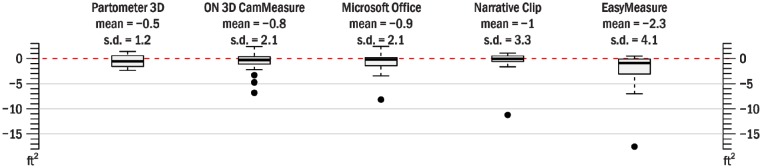
Validity of instruments as distribution of differences from actual display size

Figure 3 shows the differences between the physical measurements of the displays and the measurements obtained through each technique. The apps and methods are arranged from least to most variance (left to right). Though most are small, all of the mean and median differences are negative, indicating that display size was typically underestimated. Partometer 3D gave the closest estimates, and EasyMeasure gave the most varied.

**Figure 4.**
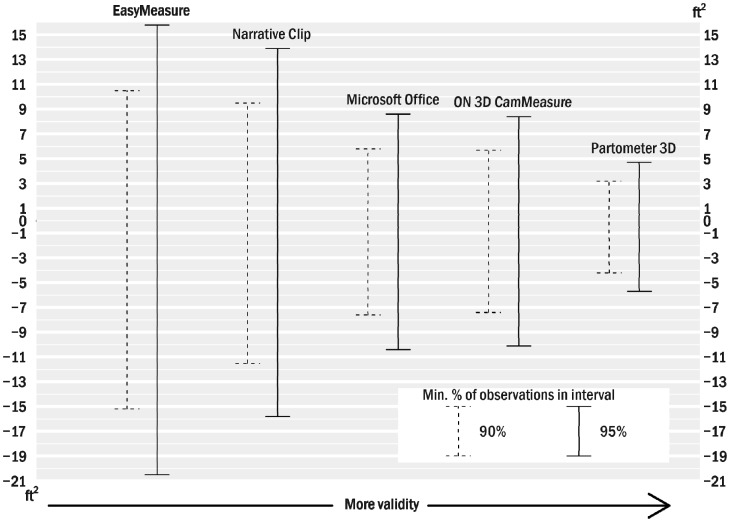
Predictions of accuracy for instruments

Using the standard deviation and mean of each instrument's differences from the actual measurement from Figure 3, the minimum percentage of measurements within a certain range was calculated using a predetermined level of confidence as defined by Chebyshev's Inequality[26]. Figure 4 shows the predicted accuracy of each instrument. The dashed and solid lines respectively show the range (in square feet) in which at least 90% and 95% of observations will fall. The Partometer 3D was the most accurate instrument from the trials, with at least 90% of its measurements falling within about 3.5 square feet of the true measurement, and at least 95% of its measurements falling within about five feet of the true measurement. The Narrative Clip and EasyMeasure were the least accurate, with 95% of observations falling within approximately 15 and 20 square feet of the actual measurement respectively.

**Figure 5.**
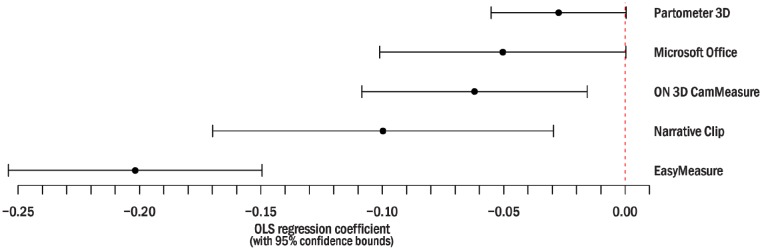
Impacts of display size on accuracy of instrument

Throughout the pilot trials of each instrument, it became evident that the size of the displays influenced the measurements for some instruments. Specifically, as the size of the display increased, the accuracy of calculations decreased and measurements were underestimated. Coefficients for each instrument from ordinary least squares regression of the actual measurements on the estimates show statistically significant differences for three instruments (Figure 5). For a one square foot increase in display size, ON 3D CamMeasure underestimates the size by 5% (coefficient of 0.05), the Narrative Clip by 10%, and EasyMeasure by 20%. The coefficients for Partometer 3D and Microsoft Office were not statistically significant, indicating that measurements were essentially unaffected by display size for these two instruments.

### 4.3. Reliability

**Figure 6.**
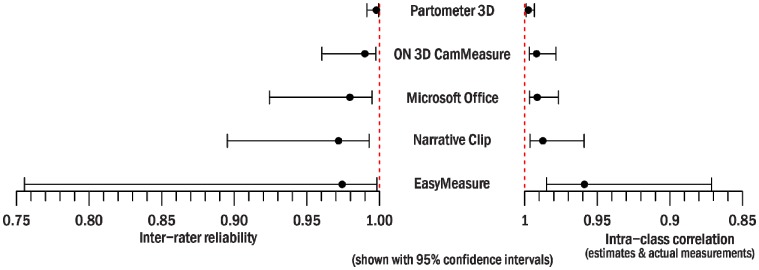
Reliability of estimates

Inter-rater reliability was calculated using a two-way absolute agreement model to determine whether systematic differences exist between the measurements of the two testers for each instrument. Figure 6 (left) shows that the lower bound of inter-rater reliability for Partometer 3D, ON 3D CamMeasure, and Microsoft Office was sufficiently high (> 0.90). Only EasyMeasure and the Narrative Clip show lower bounds that might call into question the interchangeability of users. Intra-class correlations (ICCs) were also computed between the results from each instrument and the actual (tape) measurements of the displays. Figure 6 (right) shows that acceptable lower bounds at 95% confidence were found for all instruments except for the EasyMeasure, for which the lower bound ICC fell below 0.90.

## 5. Discussion

**Figure 7.**
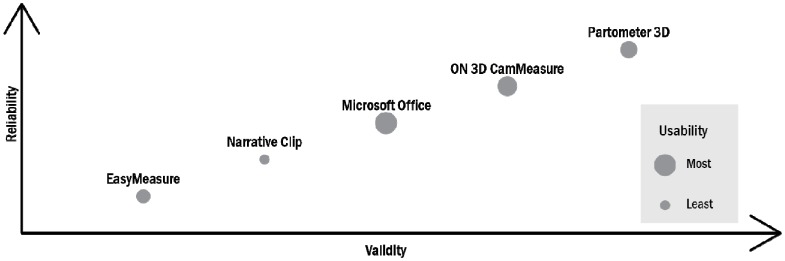
Overall results of test instruments

This paper highlights results from an assessment of existing and emerging technologies and provides the first comprehensive review of their application to tobacco product display measurement in the retail environment. As summarized in Figure 7, Easy Measure and the Narrative Clip were the least valid, reliable, and usable instruments. Partometer 3D and ON 3D CamMeasure, though taking more time and skill, produced more valid and more reliable estimates of the display sizes in the trials. The ability to manipulate images and measurements on a computer monitor (rather than a handheld device screen) made Microsoft Office the most user-friendly of the instruments.

Most of the instruments used here (i.e., Partometer 3D, ON 3D CamMeasure, and Microsoft Office) produced reliable measures, intra-class correlations both between users and between estimates and actual measurements were high. The Narrative Clip wearable camera proved difficult to aim and arguably too expensive for widespread use by tobacco control practitioners and partners, (though it should be recognized that the purpose of this product is not necessarily to produce exact photos of particular objects). However, mass-produced wearable cameras are the newest of the technologies tested here and still hold potential for photo-based measurements in retail settings. The EasyMeasure app differed from the other two apps in that it did not require a reference object of known size in the photo, but the ambitions of this technology may surpass the capabilities of handheld device sensors (accelerometers and gyrometers), at least for the time being.

In terms of validity of measurements, the three most reliable instruments as previously outlined also gave estimates closest to the actual measurements of displays. The Partometer 3D was the most accurate; for the trials, all of its estimates were within 2.5 square feet of actual size and 90% of estimates with this app are predicted to fall within 3.5 square feet of the physical measurements. This app and the method using Microsoft Office were the only two for which variations from physical measurements were not influenced by the size of the object, all others significantly underestimated dimensions as object size increased. This is important to note as power walls of tobacco products are often quite large.

This study does have some limitations that need to be acknowledged. First, it should be mentioned that all of the apps used for the purposes of this study come with disclaimers stating that the accuracy of measurements is not guaranteed. Second, rather than a laboratory experimental setting, trials in actual retailers would be optimal, however gaining permission and behind-the-counter access from owners and managers presents its own challenges. Next, after finding accurate instruments, logistics of in-store visits will still need to be addressed and standards established, (e.g., how to measure floor-based behind-the-counter displays when photos of entire shelving units are unfeasible, and how to normalize measurements for comparison—by store size, number of cash registers, square footage of another common, perhaps healthy, product). Lastly, devices other than the four used here (Samsung Galaxy S4, iPod 4, PC-based computer, Narrative Clip), along with new apps or updated versions of those used here, may perform better or differently and further research is needed to investigate these concerns. For instance, the issue of small screen size on many mobile phones could be addressed through the use of larger tablets to ease the use of certain apps.

Additional research and trials of measurement instruments holds great potential for building evidence bases for retail policy interventions. Smartphone cameras and apps are especially promising because of their almost universal use and because of the metadata collected with every picture taken. This metadata contains not only the time and day the picture was taken but also geolocation information that could be paired with socioeconomic data from the U.S. Census at various geographic levels (e.g., tract, county, metropolitan statistical area) as well as existing databases of tobacco retail licensees that show differing densities across areas. Taken together, this information can help to bolster evidence of tobacco-related disparities in general and specifically in differential product availability in areas of lower socioeconomic status. Identification of valid and reliable instruments and methods for measuring product displays holds public health implications beyond tobacco control, for instance in alcohol, nutrition, and obesity research. Our objectives for this study include motivating other researchers to perform further tests of innovative measurement tools, and to start a practical dialogue about how best to transform anecdotal knowledge of product displays as targeted and disproportionate marketing tactics into a scientific evidence base for public policy change.

## References

[1] Federal Trade Commission (2013). Cigarette Report for 2011.

[2] Ress D (2013). Altria Restructuring Marketing Efforts.

[3] Tobacco Control Legal Consortium (2011). Restricting Tobacco Advertising: Tips and Tools.

[4] Center for Public Health Systems Science (2014). Point-of-Sale: A Tobacco Control Guide.

[5] Berman M, Miura M, Bergstresser J (2012). Tobacco Product Display Restrictions.

[6] Barbeau EM, Wolin KY, Naumova EN (2005). Tobacco advertising in communities: associations with race and class. Prev Med.

[7] Campaign for Tobacco-Free Kids (2012). Deadly Alliance: How Big Tobacco and Convenience Stores Partner to Market Tobacco Products and Fight Life-Saving Policies.

[8] Henriksen L, Schleicher NC, Feighery EC (2010). A longitudinal study of exposure to retail cigarette advertising and smoking initiation. Pediatrics.

[9] Hyland A, Travers MJ, Cummings KM (2003). Tobacco outlet density and demographics in Erie County, New York. Am J Public Health.

[10] Seidenberg AB, Caughey RW, Rees VW (2010). Storefront cigarette advertising differs by community demographic profile. Am J Health Promot.

[11] Wakefield M, Germain D, Durkin S (2006). An experimental study of effects on schoolchildren of exposure to point-of-sale cigarette advertising and pack displays. Health Educ Res.

[12] Woodruff SI, Agro AD, Wildey MB (1995). Point-of-Purchase Tobacco Advertising - Prevalence, Correlates, and Brief Intervention. Health Values.

[13] Chuang YC, Cubbin C, Ahn D, Winkleby MA (2005). Effects of neighbourhood socioeconomic status and convenience store concentration on individual level smoking. J Epidemiology Community Health.

[14] Henriksen L, Feighery EC, Schleicher NC (2008). Is adolescent smoking related to the density and proximity of tobacco outlets and retail cigarette advertising near schools?. Prev Med.

[15] Loomis BR, Kim AE, Goetz JL (2013). Density of tobacco retailers and its association with sociodemographic characteristics of communities across New York. Public Health.

[16] Henriksen L, Feighery EC, Schleicher NC (2004). Reaching youth at the point of sale: cigarette marketing is more prevalent in stores where adolescents shop frequently. Tob Control.

[17] Wakefield MA, Terry-McElrath YM, Chaloupka FJ (2002). Tobacco industry marketing at point of purchase after the 1998 MSA billboard advertising ban. Am J Public Health.

[18] Spanopoulos D, Ratschen E, McNeill A (2012). Retail price and point of sale display of tobacco in the UK: a descriptive study of small retailers. PLoS One.

[19] Lee JG, Henriksen L, Myers AE (2014). A systematic review of store audit methods for assessing tobacco marketing and products at the point of sale. Tob Control.

[20] Haffey F, Brady RR, Maxwell S (2013). A comparison of the reliability of smartphone apps for opioid conversion. Drug Saf.

[21] Kardous CA, Shaw PB (2014). Evaluation of smartphone sound measurement applications. J Acoust Soc Am.

[22] Abroms LC, Lee Westmaas J, Bontemps-Jones J (2013). A content analysis of popular smartphone apps for smoking cessation. Am J Prev Med.

[23] Narrative (2015). Narrative Clip 2.

[24] Pew Research Center (2014). Cell Phone and Smartphone Ownership Demographics.

[25] comScore (2014). comScore Reports July 2014 U.S. Smartphone Subscriber Market Share.

[26] Hardy GH, Littlewood JE, Polya G (1999). Inequalities.

